# Supplementary material for: GBPL3 localizes to the nuclear pore complex and functionally connects the nuclear basket with the nucleoskeleton in plants
Source: PLoS Biol. 2022 Oct 21;20(10):e3001831. doi: 10.1371/journal.pbio.3001831 (PMC9629626; doi:10.1371/journal.pbio.3001831)
Supplement: S1 Fig — (A) GBPL3 is significantly enriched in the NE fraction compared to the total MM fraction. Data were retrieved from previously published subtractive proteomics data (PXD015919). Student t test was performed using transformed and averaged PSM values. (B) Phylogenetic analysis of GBPL3 and its homologs from various plant and animal species. The tree and bootstrap values on each branch were generated by MEGA-X using the maximum likelihood method with 100 bootstraps. Sequences used for the phylogenetic analysis can be found in S1 Data. (C) Structural similarity prediction of GBPL3 using SWISS-MODEL (https://swissmodel.expasy.org). (D) Immunoblots with an anti-GFP antibody using total protein extract from wild-type non-transformants and 2 pGBPL3-mEGFP-GBPL3 transgenic seedlings expressing representative higher and lower levels of GBPL3. Ten 1-week-old T3 seedlings were pooled for protein extraction. (E) Fluorescence imaging showing subcellular localization of GBPL3 protein in root cells. One-week-old pGBPL3- mCherry-GBPL3 transgenic plants with higher expression (upper panel) and lower expression (lower panel) levels are used for imaging. Bars = 10 μm. (F) Quantification of GBPL3-GFP immunogold labeling in the low expression line (#6) and WT non-transformant samples. The relative gold density at the nuclear membrane, the nucleoplasm, and the cytoplasm is shown (left). The percentage of gold particles around the nuclear membrane was further quantified in 3 regions: the NPC, the INM, and the ONM (right). For both WT and the GBPL3-GFP line, 3 root samples with 20 cells and approximately 300 gold particles from each were used for the quantification. All underlying data in S1 Fig can be found in S5 Data. INM, inner nuclear membrane; MM, microsomal membrane; NE, nuclear envelope; NPC, nuclear pore complex; ONM, outer nuclear membrane; PSM, peptide-spectrum match. (PDF) [file pbio.3001831.s001.pdf]

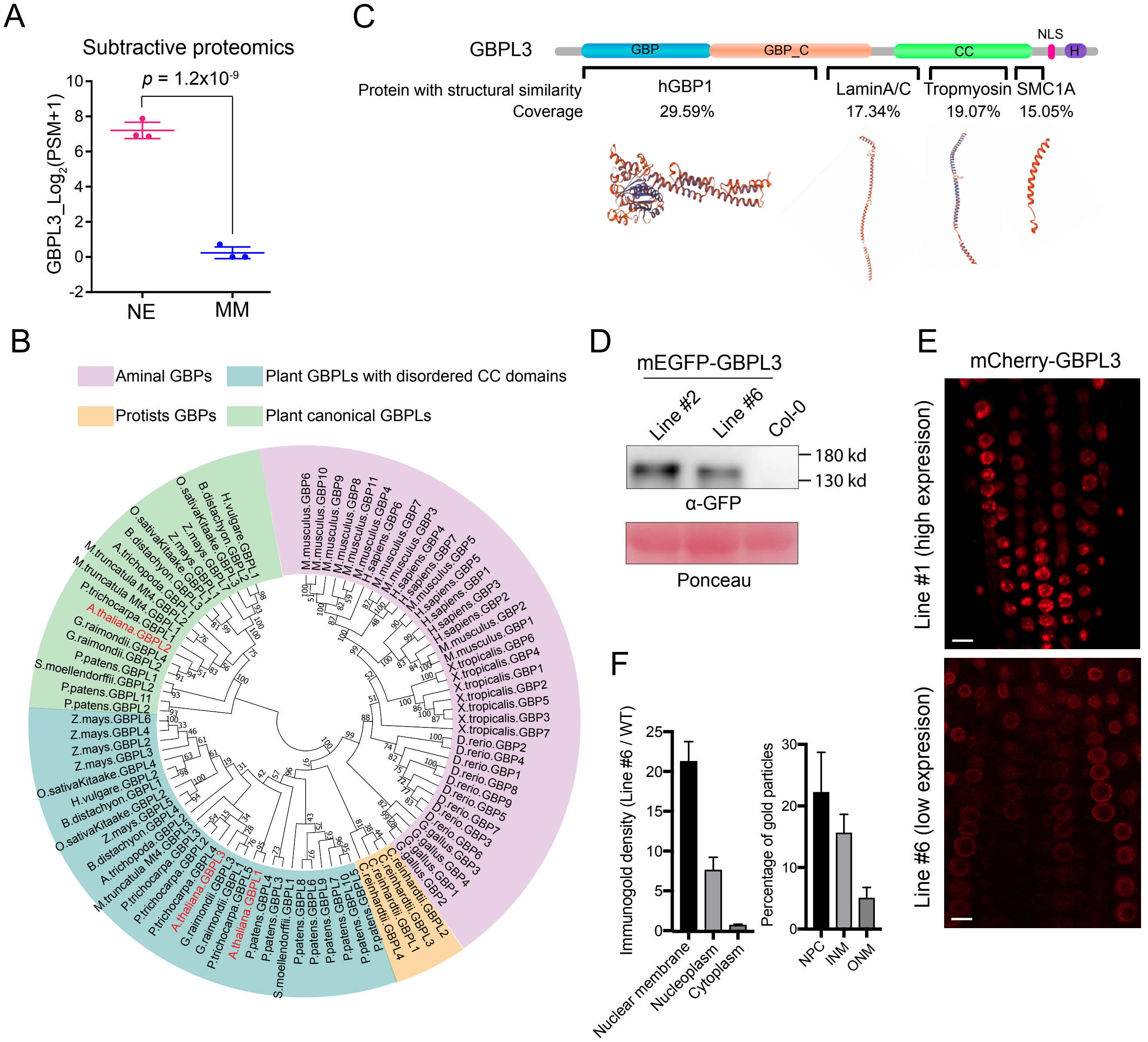

S1 Fig. Identification of GBPL3 as a nuclear envelope protein candidate

(A) GBPL3 is significantly enriched in the nuclear envelope (NE) fraction compared to the total microsomal membrane (MM) fraction. Data were retrieved from previously published subtractive proteomics data (PXD015919). Student's *t*-test was performed using transformed and averaged PSM values.

(B) Phylogenetic analysis of GBPL3 and its homologs from various plant and animal species. The tree and bootstrap values on each branch were generated by MEGA-X using the maximum likelihood method with 100 bootstraps. Sequences used for the phylogenetic analysis can be found in S1 Data.

(C) Structural similarity prediction of GBPL3 protein using SWISS-MODEL (https://swissmodel.expasy.org).

(D) Immunoblots with an anti-GFP antibody using total protein extract from wild-type non-transformants and two *pGBPL3-mEGFP-GBPL3* transgenic seedlings expressing representative higher and lower levels of GBPL3. Ten one-week-old T3 seedlings were pooled for protein extraction.

(E) Fluorescence imaging showing subcellular localization of GBPL3 protein in root cells. One-week-old *pGBPL3- mCherry-GBPL3* transgenic plants with higher expression (upper panel) and lower expression (lower panel) levels are used for imaging. Bars = 10 μm.

(F) Quantification of GBPL3-GFP immunogold labeling in the low expression line (#6) and WT non-transformant samples. The relative gold density at the nuclear membrane, the nucleoplasm, and the cytoplasm is shown (left). The percentage of gold particles around the nuclear membrane was further quantified in three regions: the nuclear pore complex (NPC), the inner nuclear membrane (INM), and the outer nuclear membrane (ONM) (right). For both WT and the *GBPL3-GFP* line, three root samples with 20 cells and ~300 gold particles from each were used for the quantification. All underlying data in S1 Fig can be found in S5 Data.
