# Supplementary material for: GBPL3 localizes to the nuclear pore complex and functionally connects the nuclear basket with the nucleoskeleton in plants
Source: PLoS Biol. 2022 Oct 21;20(10):e3001831. doi: 10.1371/journal.pbio.3001831 (PMC9629626; doi:10.1371/journal.pbio.3001831)
Supplement: S2 Fig — (A) GUS staining of transgenic Arabidopsis plants harboring the pGBPL3:GUS construct. A 5-day-old seedling, the fifth and 10th rosette leaf of a 4-week-old plant, stem, and silique are shown on the upper panel from left to right, and flower and anther tissues are shown on the lower panel. Bars = 1 cm. (B) Immunoblots with HRP-conjugated streptavidin and anti-HA antibody. Wild-type and transgenic seedlings expressing GBPL3-TurboID-3HA were treated with 50 μm free biotin for 4 h before total protein extraction (Input). The total protein was then AP with streptavidin-coated beads to enrich biotinylated protein. Two biological replicates were shown. The asterisks represent naturally biotinylated proteins. AP, affinity purified. (PDF) [file pbio.3001831.s002.pdf]

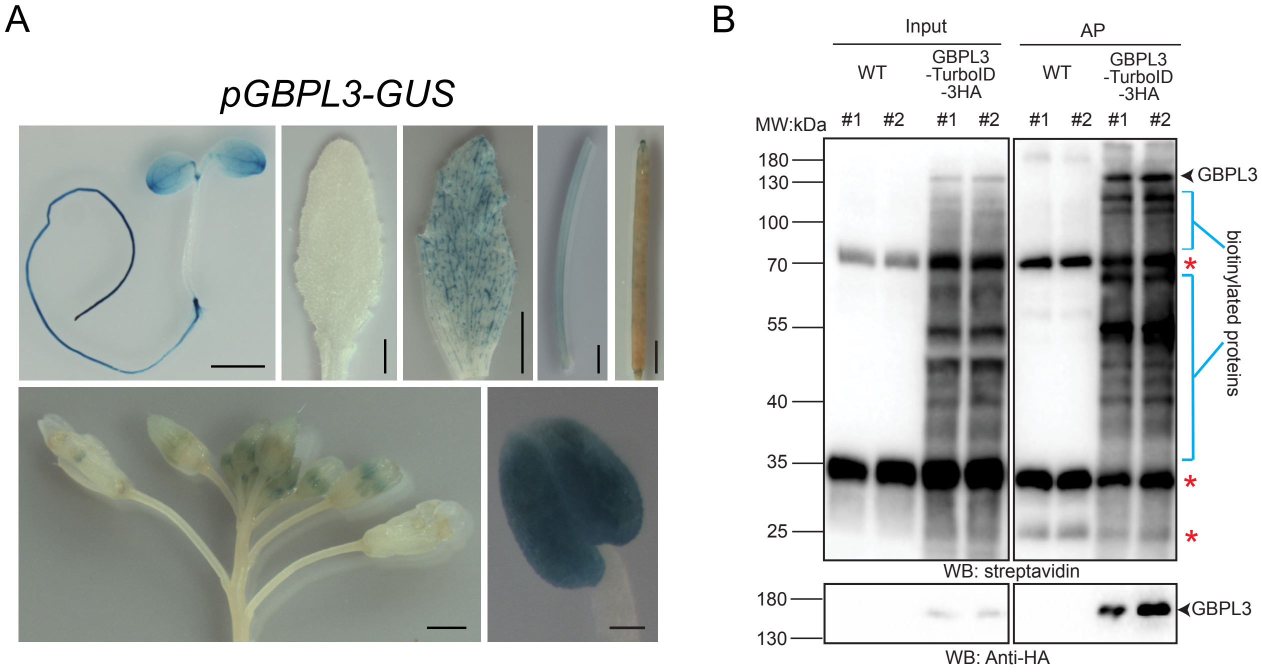

S2 Fig. Expression pattern of *GBPL3* and proximity labeling proteomics using GBPL3 as bait

(A) GUS staining of transgenic Arabidopsis plants harboring the *pGBPL3:GUS* construct. A 5-day-old seedling, the fifth and tenth rosette leaf of a 4-week-old plant, stem, and silique are shown on the upper panel from left to right, and flower and anther tissues are shown on the lower panel. Bars = 1 cm.

(B) Immunoblots with HRP-conjugated streptavidin and anti-HA antibody. Wild-type and transgenic seedlings expressing GBPL3-TurboID-3HA were treated with 50 μM free biotin for 4 hours before total protein extraction (Input). The total protein was then affinity purified (AP) with streptavidin-coated beads to enrich biotinylated protein. Two biological replicates were shown. The asterisks represent naturally biotinylated proteins.
