# Supplementary material for: GBPL3 localizes to the nuclear pore complex and functionally connects the nuclear basket with the nucleoskeleton in plants
Source: PLoS Biol. 2022 Oct 21;20(10):e3001831. doi: 10.1371/journal.pbio.3001831 (PMC9629626; doi:10.1371/journal.pbio.3001831)
Supplement: S3 Fig — (A) Heatmap showing normalized and averaged PSM values of transcription-related and mRNA processing-related GBPL3 proximal proteins from proximity labeling proteomics using GBPL3 and Nup82 as bait. Underlying data can be found in S5 Data. (B) Prediction of IDR in transcription-related and mRNA processing-related GBPL3 proximal proteins. IDRs predicted by D2P2 (https://d2p2.pro/) and by PONDR (http://www.pondr.com) are shown in the box and schematic diagram, respectively, for each protein. IDR, intrinsically disordered region; PSM, peptide-spectrum match. (PDF) [file pbio.3001831.s003.pdf]

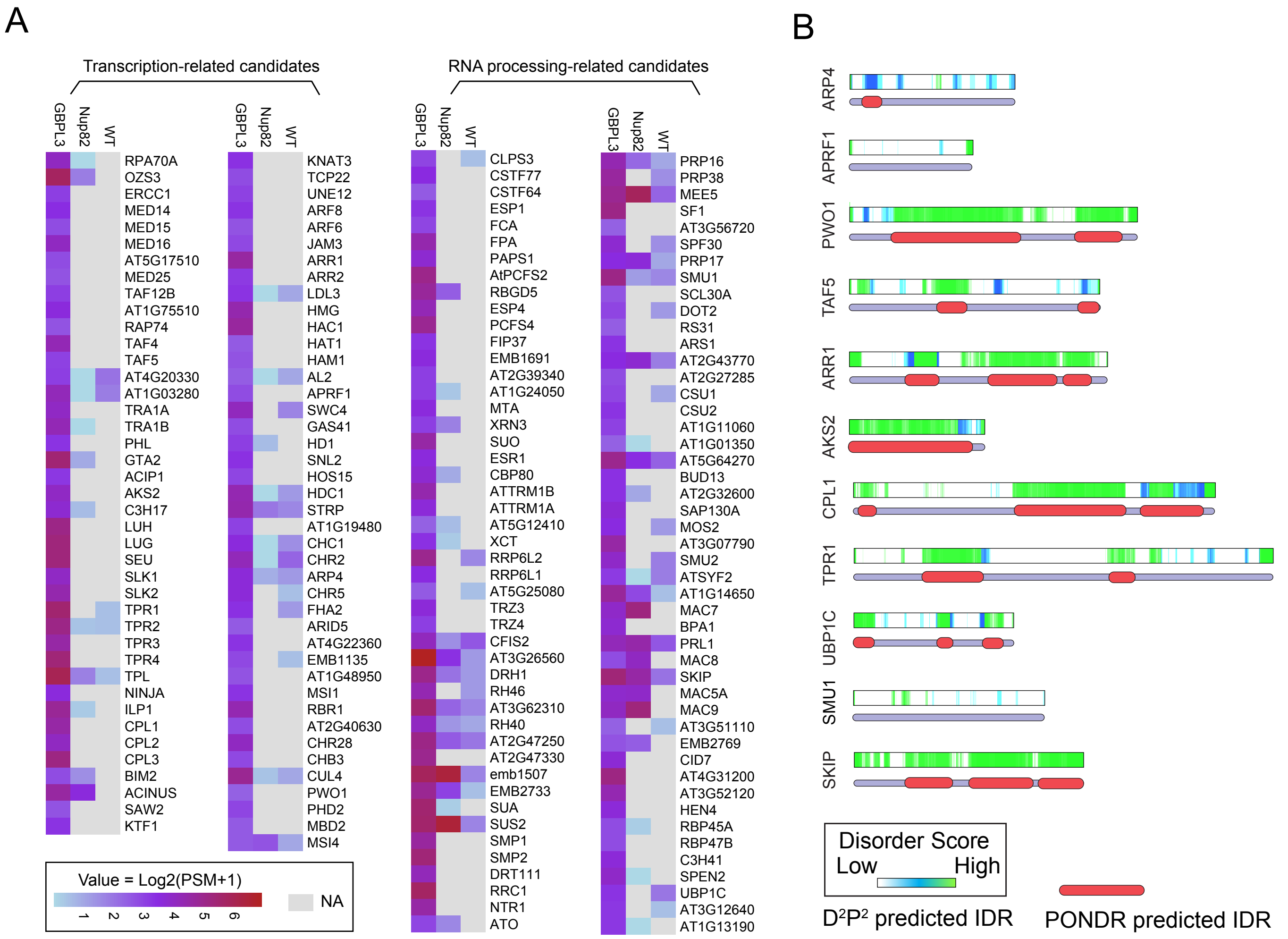

S3 Fig. GBPL3 is associated with transcription regulators and RNA processing machinery

(A) Heatmap showing normalized and averaged PSM values of transcription-related and mRNA processing-related GBPL3 proximal proteins from proximity labeling proteomics using GBPL3 and Nup82 as bait. Underlying data can be found in S5 Data.

(B) Prediction of intrinsically disorder region (IDR) in transcription-related and mRNA processing-related GBPL3 proximal proteins. IDRs predicted by D^2P^2 (https://d2p2.pro/) and by PONDR (http://www.pondr.com) are shown in the box and schematic diagram, respectively, for each protein.
