# Supplementary material for: GBPL3 localizes to the nuclear pore complex and functionally connects the nuclear basket with the nucleoskeleton in plants
Source: PLoS Biol. 2022 Oct 21;20(10):e3001831. doi: 10.1371/journal.pbio.3001831 (PMC9629626; doi:10.1371/journal.pbio.3001831)
Supplement: S4 Fig — (A) The insertion position of the 3 gbpl3 T-DNA lines. T-DNA insertions in SAIL_635_G05 and gbpl3-3 were located at the 5′ UTR of GBPL3 gene, and gbpl3-4 mutant contains a T-DNA insertion at the 3′ UTR. Arrows indicate qPCR primers. (B) Three-week-old soil-grown WT and gbpl3 T-DNA insertion mutant (SAIL_635_G05, gbpl3-3, and gbpl3-4) plants. (C) Relative expression level of GBPL3 in WT and the 3 gbpl3 T-DNA insertion mutant lines. RT-qPCR was performed using both 5-day-old whole seedlings and the 10th leaves from 4-week-old plants. Data were presented as means ± SD (n = 3 biological replicates). Actin was used as the reference gene. Student t test was performed using WT as control. **p-value < 0.01; ns stands for not significant. (D) PAM sequences of gRNAs and sequencing results showing CRISPR/Cas9-directed editing in gbpl3 mutant lines. (E) Complementation of the gbpl3-7 mutant by expressing native promoter-driven mEGFP-GBPL3 or mCherry-GBPL3 (T3 generation). One-week-old seedlings are shown. Bars = 1 cm. (F) Relative expression levels of immune-related and ion/nutrient-related maker gene in WT and gbpl3-6 measured by RT-qPCR (n = 2 biological replicates). Expression level was normalized to that in WT. Student t tests were performed (*p < 0.05, ***p < 0.001, ****p < 0.0001). ns indicates not significant. (G) The overlaps of up-regulated and down-regulated DEGs of crwn1 crwn2 (GSE106615) and gbpl3-7 (GSE199667). The significance level of the overlap was measured using hypergeometric tests. The list of overlapping DEGs was provided in S3 Data. All underlying data in S4 Fig can be found in S5 Data. DEG, differentially expressed gene. (PDF) [file pbio.3001831.s004.pdf]

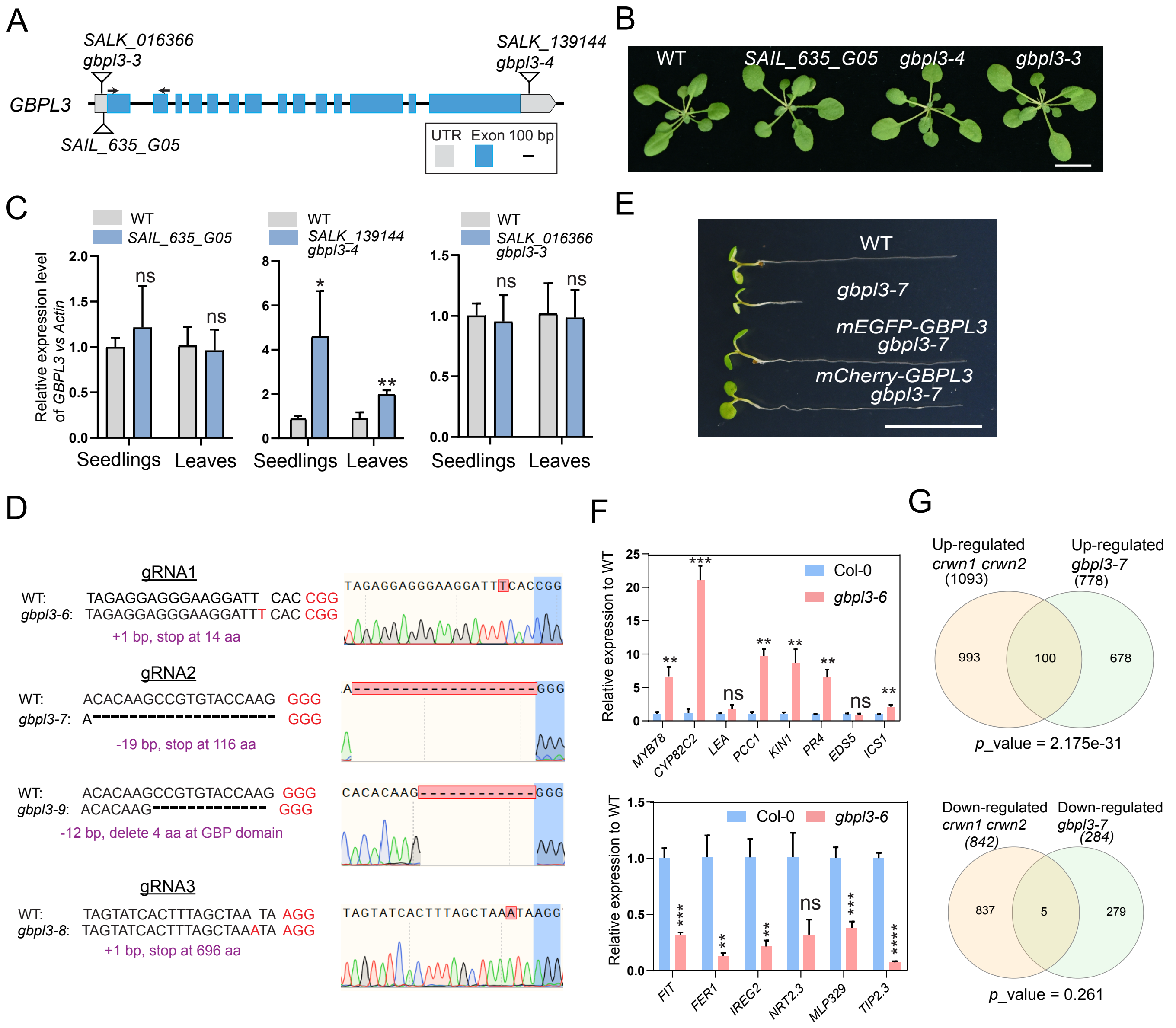

S4 Fig. Loss of GBPL3 restricts plant growth and activates stress responses in Arabidopsis

(A) The insertion position of the three *gbpl3* T-DNA lines. T-DNA insertions in SAIL_635_G05 and *gbpl3-3* were located at the 5' UTR of *GBPL3* gene, and *gbpl3-4* mutant contains a T-DNA insertion at the 3' UTR. Arrows indicate qPCR primers.

(B) Three-week-old soil-grown WT and *gbpl3* T-DNA insertion mutant (SAIL_635_G05, *gbpl3-3*, and *gbpl3-4*) plants.

(C) Relative expression level of *GBPL3* in WT and the three *gbpl3* T-DNA insertion mutant lines. RT-qPCR was performed using both 5-day-old whole seedlings and the 10th leaves from 4-week-old plants. Data were presented as means ± SD (n = 3 biological replicates). Actin was used as the reference gene. Student's *t*-test was performed using WT as control. $^{**}p$-value < 0.01. ns stands for not significant.

(D) PAM sequences of gRNAs and equencing results showing CRISPR/Cas9-directed editing in *gbpl3* mutant lines.

(E) Complementation of the *gbpl3-7* mutant by expressing native promoter-driven *mEGFP-GBPL3* or *mCherry-GBPL3* (T3 generation). One-week-old seedlings are shown. Bars = 1 cm.

(F) Relative expression levels of immune-related and ion/nutrient-related marker genes in WT and *gbpl3-6* measured by RT-qPCR (n = 2 biological replicates). Expression level was normalized to that in WT. Student's *t*-tests were performed ($^{*}p < 0.05$, $^{***}p < 0.001$, $^{****}p < 0.0001$). ns indicates not significant.

(G) The overlaps of up-regulated and down-regulated DEGs between *crwn1 crwn2* (GSE106615) and *gbpl3-7* (GSE199667). The significance level of the overlap was measured using hypergeometric tests. The list of overlapping DEGs was provided in S3 Data. All underlying data in S4 Fig can be found in S5 Data.
