# Supplementary figures and images for: GBPL3 localizes to the nuclear pore complex and functionally connects the nuclear basket with the nucleoskeleton in plants
Source: PLoS Biol. 2022 Oct 21;20(10):e3001831. doi: 10.1371/journal.pbio.3001831 (PMC9629626; doi:10.1371/journal.pbio.3001831)

**S1D Fig**

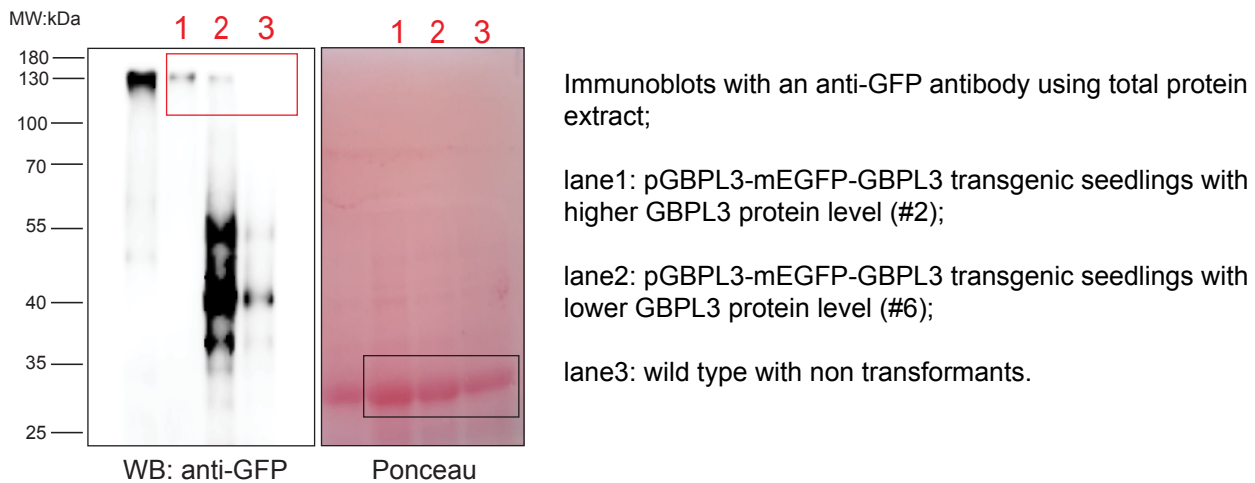

**S2B Fig**

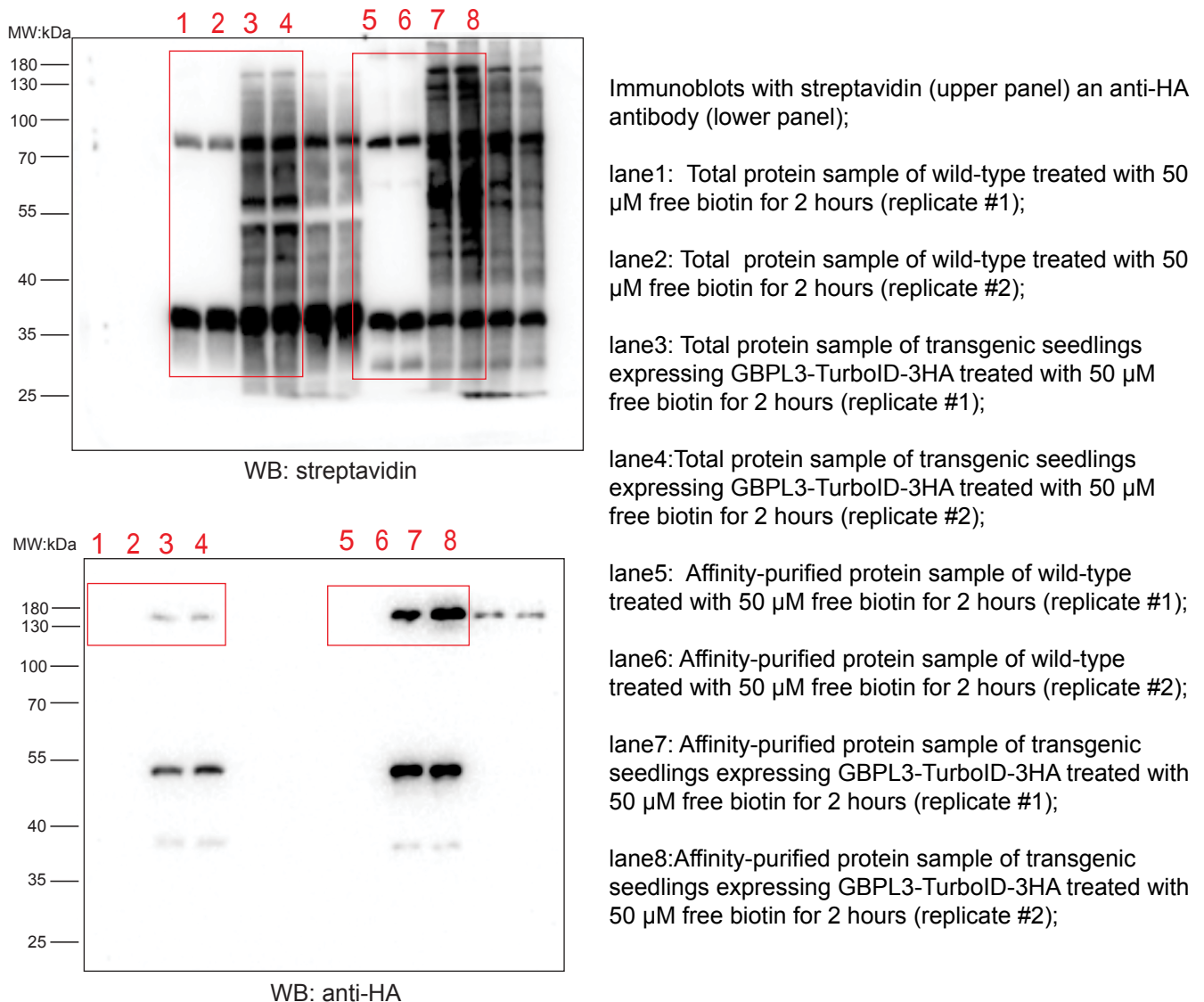

**S1 Raw Images. Raw images.**

Supplement: S1 Raw Images — (PDF) [file pbio.3001831.s010.pdf]
