# Supplementary material for: GBPL3 localizes to the nuclear pore complex and functionally connects the nuclear basket with the nucleoskeleton in plants
Source: PLoS Biol. 2022 Oct 21;20(10):e3001831. doi: 10.1371/journal.pbio.3001831 (PMC9629626; doi:10.1371/journal.pbio.3001831)
Supplement: Attachment — Submitted filename: Response to reviewers.pdf [file pbio.3001831.s011.pdf]

Reviewers' comments / Authors' response

Rev. 1:

This works builds from the author's previous work (Tang et al, 2020; Tang et al, 2022), identifying components of the Arabidopsis nuclear pore complex by proteomics. In these studies, the protein GBPL3 was discovered; characterization of this newly discovered component of the Arabidopsis nuclear pore complex is the subject of this current manuscript. GBPL3 recruits chromatin remodelers to the NPC, suggesting a role for transcriptional regulation. GBPL3 further interacts with the nucleoskeleton. This work is in contrast with a recent publication (Huang et al., 2021) suggesting that GBPL3 acts as a transcriptional regulator of immune response within the nucleus.

Overall, I found the data to be compelling, claims appropriately backed with data, and I particularly appreciate the caveats and alternative explanations provided by the authors. Other than the two typos listed below, I have no concerns about the data presented in this manuscript and feel that this makes a valuable addition to our understanding of the plant nucleopore and further provides fodder for future studies.

Line 53 - "In metazoan" should be "In metazoans".
Line 76 - "CROWED" should be "CROWDED".
We thank the reviewer for recognizing the significance and quality of our work. The typos were corrected.

Rev. 2:

This is a detailed and carefully executed study which significantly enhances knowledge of the plant nuclear pore complex, by adding a new and functionally significant protein.

15 However, the functional mechanisms behind- consider rewording; 'behind' is unclear as to meaning
Revised to "the underlying molecular mechanisms are not completely understood"

18 we found that GBPL3 is localizes to the nuclear rim and is enriched in the nuclear pore.
"is" was deleted

26 bona fide - Latin; italicise.
"bona fide" Italicized

74 Nup136 and Nup82 are thought to share a common evolutionary history with a plant75 specific nucleoskeleton protein KAKU4, and conserved motifs in these three proteins mediate physical 76 interaction with CROWED NUCLEIs (CRWNs), filamentous proteins that compose the nucleoskeleton in 77 Arabidopsis (39). Please provide a reference for the common evolutionary history of KAKU4.
The reference is cited at the end of the sentence (Ref 43. Mermet S, et al (2021)).

227 formed spontaneous nuclear condensates while the rest remaining five diffuse in the nucleus

“the rest five” was replaced with “the other five”.

231 under the transient overexpression condition better: in transient overexpression
Revised as suggested.

336- Adding more complexity, 337 although the four CRWN homologs have been shown to function redundantly in building the 338 nucleoskeleton and in regulating chromatin organization and genome activity (58-60), their double mutant 339 with gbpl3 led to distinct phenotypes, raising an intriguing hypothesis that different types of NB 340 nucleoskeleton joints can be formed by unique combinations between GBPL3 with CRWN proteins, which 341 may differentially contribute to gene expression regulation. Consider rewording the above to simplify for the reader.
The original sentence was broken into two to facilitate understanding.

Figure 1 E) Three-dimensional reconstruction of root cells expressing mEGFP-GBPL3 in a line with lower expression. Please reword for greater clarity; this is not a three dimensional reconstruction…
Thanks for pointing this out. Revised to “z-stack imaging followed by projection of root cells …”.

Rev. 3:

The manuscript from Yu Tang, Man Ip Ho, Byung-Ho Kang and Yangnan Gu entitled "GBPL3 is a novel component of the nuclear pore complex in plants and functionally connects the nuclear basket with the nucleoskeleton" is an original contribution deciphering the plant NPC structure and function. Starting from the innovative proximity labeling proteomics technique recently developed in plants by the authors, the authors here focused on a specific component called GBPL3 identified in their initial experiments on the nuclear envelope. They clearly show using bio-informatics, molecular biology techniques and genetics that GBPL3 is enriched at the NPC basket, physically interacts with lamina and NUP components, is connected to the transcription machinery and that its mutation affects plant development in an additive/synergistic genetic effect with components of the plant lamina. This manuscript provides a strong validation of the previous observations made by proximity labeling proteomics and describes a new component connected to the NPC basket. For these reasons, I strongly recommend to consider this manuscript for publication in PLOS Biology with the following minor revisions.

Minor revisions:

Title: Although there is no doubt about the fact that GBPL3 is located at the NPC basket and interacts with the lamina and NPC, it is still questionable whether it is a true component of the nuclear pore basket or rather a protein interacting preferentially with certain components of the NPC basket and connecting the NPC to the transcription machinery. I would suggest to slightly change the title.
Agreed. The title is changed to “GBPL3 localizes to the nuclear pore complex and functionally connects the nuclear basket with the nucleoskeleton in plants”.

l31: is the NPC the only possibility for a protein to access the nucleus as the ER that is in continuity to the NE can provide a way to TM anchored proteins to be imported into the nucleus.
Major models (diffusion-retention, sorting motif-mediated, and NTR-mediated) support that TM proteins are synthesized at ER and are translocated to the inner nuclear membrane via the NPC

(likely through the side pore). However, evidence supporting vesicle-mediated transport (from ONM to INM) does exist, especially during viral replication. We thus revised “sole gateway” to “main gateway” in the text.

Reference:
Katta, S et al., Trends in Cell Biology (2014) 24: 221-229

l32: This number seems a little too large as to my knowledge the NPC is made an octomeric structure with each monomer containing about 30-40 NUPs (i.e. 240-320 proteins / NPC?)
Our description in the main text is accurate. Human NPC contains ~1000 Nups and is ~110 MDa, and yeast NPC contains at least 456 Nups with a molecular weight ~50 MDa.

l37-38: I recommend to add a citation describing the Plant NPC. Fiserova and Golberg 2009 used electron microcopy to investigate the NPC structure and gave some insights about the NPC basket. They proposed that filaments at the NPC basket are about 10nm.
The filaments of the nuclear basket vary in different species. In yeasts and human, they are 60-120 nm long (Cibulka et al., 2022 and Arlucea et al., 1998). Indeed, Fiserova et al. found filaments at NPC basket are about 10 nm in onion cells and tobacco BY2 cells. To avoid making confusion on the NB filament length, we deleted the length values from the main text. Instead, Fiserova et al. (2009) and Arlucea et al. (1998) are cited for readers’ reference.

Reference:
Cibulka et al., SCIENCE ADVANCES 8, eabl6863 (2022).
Arlucea et al., JOURNAL OF STRUCTURAL BIOLOGY 124, 51–58 (1998).

l47: I suggest to refer to "gene gating" by citing the initial Blobel's paper (1985) or to a review such as Tamura 2020, Journal of Plant Research
K. Tamura et al. (2020) and G. Blobel et al. (1985) have been cited now.

l73: Can the author reformulate these two sentences? NUP82 and NUP136 are two paralogs that contain FG-repeats and are located at the NB. NUP82 and NUP136 are two plant specific proteins that are redundantly required for...
The sentences were revised to “The Arabidopsis FG-repeats containing nucleoporin Nup136 is likely a functional homolog of vertebrate Nup153 and has been reported to be involved in total mRNA export as well as microRNA biogenesis through its direct interaction with the TREX-2 complex [40, 41]. Nup82 is a paralog of Nup136 and is plant specific. Nup82 and Nup136 interact with each other in the NB and are redundantly required for activation of SA-mediated pathogen resistance in Arabidopsis [42].”

l89: Can the authors better connect this sentence with the following one?
The sentence was revised to “The *gbpl3* mutants display stunted growth and sterility.”

l107-Fig1A: can the authors specify in the Fig1A legend which control is control 1 and control2. Nup93a-BoiID2 (control1?) and YFP-BioID2 (control2?) mock-treated samples were used as controlsYFP-BioID2 mock-treated samples (control1?) and non-transformant plants (NT) (control2?) were used as controls.

The controls are now specified in the figure legend.

l123: can this be shown in a supplemental figure?
This has been added as the new Fig. S1C.

l134-135: The authors described a phenomenon that is likely due to the genomic position of the transgene insertion affecting the level of expression and/or that can be linked to a missing regulatory element in their construct. I feel that this section would need some editing to better explain the observed results.
First, the authors proposed that there are two set of lines (i.e. high vs low expression) among their transgeneic lines. Can they provide the number of lines produced and the number of high and low expressing lines in Fig S1C.
This information has been added to Method under “Plant material and growth conditions”.

Second, "Even though"... I guess the authors mean that this kind of position effects are usually observed with a p35S promoter and not/less with an endogenous promoter? can this be reformulated to help the reader to understand this unexpected result?
The sentence was revised to “Although the construct was driven by the GBPL3 native promoter, we were able to detect a difference in the mEGFP-GBPL3 expression level in independent transgenic lines based on immunoblots (Fig. S1D), potentially due to different copy number or insertion loci of the transgene.”

l159: "in vivo" in italic
Revised.

l188-189-Fig 2B: It is surprising that the images showing CRWN1 and CRWN3 show that they are not enriched at the NE as described in other studies. As this has already been described in other studies (see Dittmer et al 2007) I would suggest to remove the images of CRWN1 and CRWN3 expression. If possible, it would also be better to select sections of confocal images showing expression at the NE (i.e. a cross section of the median part of the nucleus).
CRWN1 localizes to the nuclear periphery but displays a meshwork-like pattern, which may lead to the misinterpretation of the reviewer. The nucleoplasm localization of some CRWNs (e.g. CRWN2 and CRWN3) under overexpression conditions has been documented by multiple publications, including Dittmer et al. 2007 and Sakamoto and Takagi., 2013. Therefore, our data are not inconsistent with other reports.

Reference:
Sakamoto and Takagi Plant Cell and Physiology (2013) 54: 622
Dittmer et al. The Plant Cell (2007) 19: 2793

l193-Fig 2D-E-F: For clarity; can the author provide results with CRWN2 in their transient expression assays (even if the result is negative)?
For some reasons, our CRWN2-GFP construct is not expressed at a visible level in Benthi. Despite missing of CRWN2 imaging data, the Y2H result strongly supports its physical interaction with GBPL3. Together with their genetic interaction shown in Fig. 5, CRWN2 is expected to functionally associate with GBPL3, like other CRWNs.

CRWN4 is missing in these experiments (can the authors give an explanation?).
We had technical issues with the cloning of CRWN4 cDNA, and thus requested it from another lab. Unfortunately, we were not able to confirm the full-length sequence of the requested clone using sequencing. We therefore did not include CRWN4.

l197-Fig 2F: Here again, it would be also better to select sections of confocal images showing expression at the NE (i.e. a cross section of the median part of the nucleus).
Because these condensates were predominantly distributed at the nuclear surface and relatively dynamic, using cross section will dramatically decrease the condensate number and make them less observable.

l226-230: As GBPL3 form aggregates, one possibility is that it will have the ability to spontaneously attract other co-expressed proteins. I would strongly recommend to add at least one negative control in this experiment by choosing a nucleoplasmic protein that was not suggested to interact or to be in close proximity to GBPL3.
Thanks for the suggestion. A negative control (a nucleoplasmic localized transcription factor) has been added to Fig. 3C now.

l278-281: can the author provide as a supplementary fig a venn diagram showing the overlapping between gbpl3 and crwn1 crwn2 DEGs?
can they also provide a ref to crwn1 crwn2 transcriptomic experiments (i.e. Mukulski et al 2019 and/or Choi et al 2019, ...).
The suggested data have been added to Fig. S4G, and the references were also added.

l298: CRWN1 was often associated with stronger phenotypes in earlier studies from the Eric Richard's group. These previous observations can be referred here.
We have cited Choi et al., 2019 and Sakamoto et al., 2013 as suggested.

l311-314: Maybe the authors can also recall here that in Y2H, a clear physical interaction was recorded only with CRWN2?
This sentence mainly refers to genetic interactions, and the Y2H data is particularly relevant.

l350: « in vivo » in italic
Revised.
